# Internet-Delivered Cognitive-Behavioral Therapy (iCBT) for Spanish-Speaking Adults with Prolonged Grief Disorder (PGD): A Randomized Feasibility Trial

**DOI:** 10.3390/bs15101312

**Published:** 2025-09-25

**Authors:** Cintia Tur, Daniel Campos, Laura Díaz-Sanahuja, Sara Fernández-Buendía, Jorge Grimaldos, Laura De la Coba-Cañizares, Evaldas Kazlauskas, Soledad Quero

**Affiliations:** 1 Faculty of Health Sciences, Universidad Internacional de la Rioja (UNIR), 26006 La Rioja, Spain; cintia.tur@unir.net; 2 Departamento de Psicología y Sociología, Universidad de Zaragoza, 50005 Zaragoza, Spain; camposd@unizar.es; 3 Departamento de Psicología de la Salud, Universidad de Alicante, 03690 Alicante, Spain; laura.diazsanahuja@ua.es; 4 Department of Basic, Clinical Psychology, and Psychobiology, Universitat Jaume I, 12071 Castellón, Spain; buendia@uji.es (S.F.-B.); grimaldo@uji.es (J.G.); lde@uji.es (L.D.l.C.-C.); 5 Center for Psychotraumatology, Institute of Psychology, Vilnius University, LT-01513 Vilnius, Lithuania; evaldas.kazlauskas@fsf.vu.lt; 6 CIBER de Fisiopatología de la Obesidad y Nutrición (CIBEROBN), Carlos III Institute of Health, 28029 Madrid, Spain

**Keywords:** internet-delivered cognitive-behavioral therapy, randomized feasibility trial, prolonged grief disorder, Spanish-speaking sample

## Abstract

Losing a loved one is a painful process that usually diminishes over time. Despite that, one out of ten bereaved adults are at risk of developing Prolonged Grief Disorder (PGD) Internet-delivered cognitive-behavioral therapies (iCBTs) can reach individuals in need of therapy and are both cost-effective and clinically effective. This study aimed to investigate the feasibility of an iCBT for Spanish-speaking individuals with PGD (GROw) compared to the same intervention delivered in a face-to-face videoconferencing format. As a secondary objective, the potential efficacy of GROw was explored. A total of 31 participants were randomized to the experimental group (N = 16) (GROw) and the active control group (N = 15) (videoconferencing treatment). There were four assessment points: baseline, after intervention, and 3- and 12-month follow-ups. Both GROw and videoconferencing treatments were well accepted in terms of preferences, expectations, satisfaction and evaluation about the usefulness of the intervention, and showed significant symptomatology reduction with large effect sizes in most of the outcomes. The dropout rate was 50% in the GROw and 33.33% in the videoconferencing group. GROw is a feasible, well-accepted iCBT for the treatment of PGD with promising results related to its potential efficacy.

## 1. Introduction

Losing a loved one is a painful process that is unique to each individual (Boelen & Smid, 2017; Stroebe et al., 2017) and has psychological, physical and social ramifications (Shear, 2015a). Feelings of regret and longing are considered natural during the experience of losing a loved one to death and usually lessen over time (Jordan & Litz, 2014). However, a percentage of grievers develop long-term and disturbing reactions that interfere with their life. Lundorff et al. (2017) found that one in ten bereaved adults worldwide is at risk for long-term and disturbing grief.

Prolonged Grief Disorder (PGD) is defined by the International Classification of Diseases (ICD-11) of the World Health Organization (World Health Organization, 2018) as a persistent and pervasive grief response characterized by longing or persistent preoccupation with the deceased, accompanied by intense emotional pain (e.g., sadness, guilt, or anger). The response clearly exceeds expected social, cultural, or religious norms for the individual’s culture and context and causes significant impairment. Diagnosis of PGD requires at least persistence of symptoms for 6 months after the loss.

PGD has multiple consequences for individuals that could impact their quality of life, including worsening of physical health and somatic problems (Cunningham et al., 2025). One of the factors that has made PGD a major public health concern worldwide in the last few years is the SARS-CoV-2 coronavirus (COVID-19) (Eisma et al., 2020). According to the prevalence of disturbing and disabling grief related to deaths caused by COVID-19, recent studies found that over one-third of relatives developed PGD (Tang & Xiang, 2021). However, findings from the studies on COVID-19-related grief also reveal that higher grief severity was related to unnatural causes of death during the pandemic, rather than COVID-19-related deaths (Killikelly et al., 2025). Living the grieving process in isolation and the circumstances related to a death during the pandemic were associated with the development of mental health problems (e.g., Diolaiuti et al., 2021; Kokou-Kpolou et al., 2020).

Group (e.g., Chow et al., 2019; Harrsen et al., 2024; Lacasta & Cruzado, 2023; Wenn et al., 2019) and individual interventions (e.g., Acierno et al., 2021; Buck et al., 2020; Boelen et al., 2007; Killikelly et al., 2025; Shear et al., 2016) have been developed to treat and prevent PGD with good results, reducing PGD symptoms in bereaved adults (Bergman et al., 2017; Johannsen et al., 2019; Wittouck et al., 2011). However, even in high-income countries (Chisholm et al., 2016), most individuals who need treatment do not receive any services due to various barriers, such as socioeconomic or geographic ones (Borson et al., 2019; López-Lara et al., 2012), and the number of individuals affected by mental disorders is large and growing (Harvey & Gumport, 2015; Kessler et al., 2005).

Internet-delivered cognitive-behavioral therapy (iCBTs) allows reaching more people who need treatment. These types of self-applied treatments (i.e., without a psychotherapist but with the specific content on a website) are cost-effective (Rohrbach et al., 2023) and constitute a well-established therapeutic format showing a clinical efficacy similar to that of face-to-face therapy (Hedman-Lagerlöf et al., 2023).

There are some internet and computer-based interventions (e.g., Brodbeck et al., 2019; Eisma et al., 2015; Kaiser et al., 2022; Lenferink et al., 2023; Litz et al., 2014; Reitsma et al., 2023; Treml et al., 2021; Wagner et al., 2022) for treating and preventing PGD with promising results (Finucane et al., 2024; Wagner et al., 2020; Zuelke et al., 2021). However, as far as we know, only one study is known to have tested an iCBT for a Spanish-speaking population. The Randomized Control Trial (RCT) of Dominguez-Rodriguez et al. (2023) was conducted with 114 adults from Mexico who lost a loved one during the COVID-19 pandemic. Participants were randomly assigned to an intervention group or to a waiting list. The intervention consisted of a self-applied and web-based CBT treatment and the results showed that it was effective in reducing bereavement symptoms at post-treatment and follow-up. Despite the relevance of this study, as far as we know no study has evaluated the feasibility of an iCBT in the Spanish population.

The main aim of this study was to examine the feasibility of an iCBT for the Spanish population with PGD (GROw) compared to the same intervention delivered in a videoconferencing format for PGD. Specifically, the objectives were (1) to assess patients’ opinion of GROw and the videoconferencing intervention as a treatment for PGD (preferences and opinions when comparing the two types of intervention formats, expectations, satisfaction, and participants’ evaluation about the usefulness of the intervention); (2) to evaluate the feasibility of recruiting the target population; and (3) estimate recruitment and retention rates to guide the planning of a future large-scale RCT. As a secondary aim, the study also explored the potential effectiveness of GROw, considering both within-group changes (pre- to post-treatment) and between-group differences in PGD-related symptoms.

## 2. Materials and Methods

### 2.1. Study Design

A two-arm randomized trial study of an iCBT intervention for PGD was conducted. After signing the informed consent, participants were randomly assigned to two conditions with the same therapeutic content but using different formats: (1) experimental group (iCBT: GROw) and (2) active control group (videoconferencing intervention). The diagnosis of PGD was based on two conditions: (1) a score ≥ 25 on the 19-item Inventory of CG (ICG) (Limonero et al., 2009; Prigerson et al., 1995) and (2) diagnosis of PGD based on an interview based on the ICD-11 criteria. This study was approved by the Ethical Committee of the Universitat Jaume I (Castellón, Spain) (file number CD/002/2019, 6 March 2019) and registered on the clinicaltrials.gov (NCT04462146, 8 July 2020). The study is reported following the Consolidated Standards of Reporting Trials of Electronic and Mobile Health Applications and online telehealth guidelines (Eysenbach & CONSORT-EHEALTH Group, 2011) and the extension of the Consolidated Standards of Reporting Trials (CONSORT) statement for pilot and feasibility studies (Eldridge et al., 2016). According to the CONSORT statement for a feasibility study (Eldridge et al., 2016) a formal sample size calculation is not required. However, a sample of 48 people (24 per group) was proposed in the previously published protocol of this study (Tur et al., 2021) based on an estimated number of dropouts of 30% (Andrews et al., 2010; Rachyla et al., 2020; Van Ballegooijen et al., 2014) for the secondary objective of this study (to explore the potential efficacy of GROw). Finally, 31 participants were recruited to this study.

Participants received reminder emails about assigned assessments during treatment to promote participant retention. The assessment points established previously in the protocol were: baseline (t1), immediately after the intervention (t2), 3-month follow-up (t3), and 12-month follow-up (t4). However, compared to the published study protocol (Tur et al., 2021), several changes to the study design must be noted. First, potential efficacy was explored using a mixed-model analysis approach for intent-to-treat (ITT) data. Second, due to the high dropout rates observed during the study, follow-up measures were not included in the analyses. The significant loss of participants during the follow-up period made it difficult to ensure the reliability and validity of the results, leading to the exclusion of these data from the analysis reported in the present study. Finally, the objective of exploring participants’ opinions on the study design, materials, and assessments was removed, as the reduced sample size would not provide sufficient power to draw meaningful conclusions.

### 2.2. Recruitment, Screening, and Eligibility Criteria

Individuals who expressed interest in the study were reached via a project-specific email address and/or telephone line. Recruitment strategies included the use of general social media platforms (such as Facebook, Twitter, and Instagram), professional networks (e.g., LinkedIn), local media outlets (including newspapers, radio programs, and podcasts), as well as posters displayed in various locations at Universitat Jaume I and Universitat de València. Additionally, participants were recruited through the Emotional Disorders Clinic at Universitat Jaume I. To verify eligibility, trained therapists conducted telephone interviews to assess whether potential participants met the study’s inclusion and exclusion criteria. Inclusion criteria were as follows: (1) age ≥ 18 years; (2) meeting diagnostic criteria for ICD-11 PGD (World Health Organization, 2018) and having a total score of >25 on the 19-item inventory of Complicated Grief (ICG) (Limonero et al., 2009; Prigerson et al., 1995); (3) understanding spoken and written Spanish; (4) email access; (5) having basic computer literacy and access to computer and internet; and (6) signing an informed consent form. Given that GROw is not culturally adapted to other countries, an additional inclusion criterion that was not proposed in the published study protocol (Tur et al., 2021) was included: (8) residency in Spain or being of Spanish nationality and living abroad in another country. Exclusion criteria were as follows: (1) suicide risk or self-destructive behaviors; (2) presence of another severe mental disorder (e.g., substance abuse or dependence, psychotic disorder, dementia, borderline personality disorder) or medical condition whose severity or characteristics prevent participation in treatment; (3) receiving another psychological treatment during the study; (4) an increase and/or change in the medication during the study. The data resulting from individuals who increased and/or changed their medication during the study period (from baseline to post-intervention) were excluded from the statistical analyses in this study. During the screening phone call, participants were informed of the study conditions (duration, format conditions, etc.). Participants who met the criteria were randomized to the two conditions (GROw and videoconferencing treatment) by an independent researcher using EPIDAT V.4.2. (Consellería de Sanidade, Xunta de Galicia, Spain). Participants agreed to the study voluntarily, did not receive financial compensation for participating, and were able to leave the study at any time. All participants were assigned to the assessments through phone call interviews and a specific online platform: https://psicologiaytecnologia.labpsitec.es/ (accessed on 15 July 2025).

### 2.3. Intervention

#### 2.3.1. Therapeutic Components

Both groups (experimental and control) received the same therapeutic components in a different format. The main components were adapted from Neimeyer’s program for reconstructing the meaning of loss during complicated grief (Botella et al., 2008; Neimeyer, 2000, 2001) and include components of Complicated Grief Treatment by Shear (2015b) (i.e., memories form, imaginary conversation with the deceased person), elements from other computerized psychological treatments (i.e., exercises for cognitive reappraisal) (Hoffmann et al., 2018; Wagner et al., 2006; Wagner & Maercker, 2007), and mindfulness activities and compassion and self-compassion strategies (Campos et al., 2019; García-Campayo et al., 2016; García-Campayo, 2019; García-Campayo, 2018; Lopez-Montoyo et al., 2019; Navarro-Gil et al., 2020). The treatment was designed and adapted from previous studies by a team of clinical psychologists with experience in trauma, stress-related disorders, and PGD. It is composed of different therapeutic components: motivation to change, psychoeducation, behavioral activation, experiential exposure, mindfulness and compassion strategies, writing assignments for reconstructing the meaning of loss, cognitive reappraisal, and relapse prevention. More information about the therapeutic components of the intervention can be found in Tur et al. (2021).

#### 2.3.2. iCBT for PGD (GROw)

**Figure 1:**
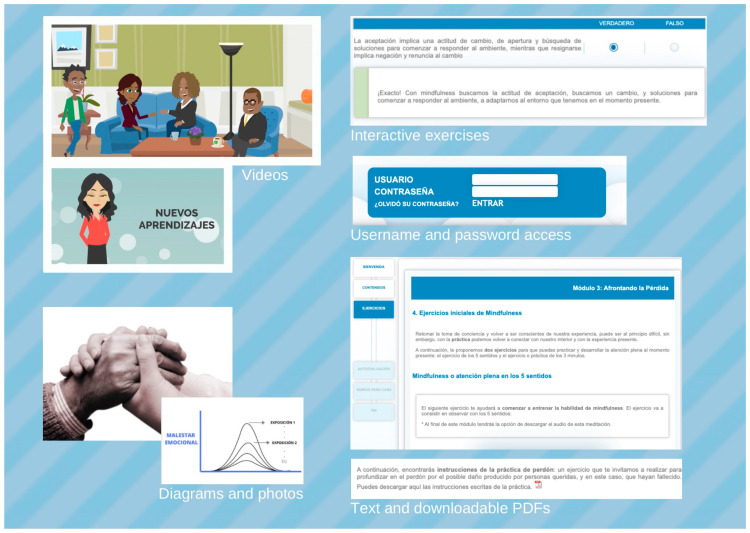
“Screenshots” of the “Psychology and Technology” web platform.

GROw is an individual self-applied program accessed with website that requests a username and password: https://psicologiaytecnologia.labpsitec.es (accessed on 15 July 2025) (see Figure 1). Participants can log in to the program at any time to see new content, review past content, see the calendar with the record of the sessions, and observe their progress through visual graphs (i.e., measures of grief, depression, anxiety and positive and negative affect). Therapeutic contents are sequentially organized into 8 modules (estimated to last approximately 60 min each). Participants were instructed to complete one module per week, being able to extend modules 4, 5 or 6 (dedicated to writing assignments for reconstructing the meaning of loss) for two weeks due to its complexity and length. The estimated extension to carry out the treatment was 8–10 weeks. The GROw program contains videos, texts, audios, photos, downloadable pdfs, personalized diagrams, and self-assessment questions about the therapeutic content (see Figure 1). In addition, to access the platform, participants received a brief weekly phone call (10–15 min) from a therapist (always from the same assigned trained therapist). The objectives of the phone calls were: (1) to motivate the participants to continue with the program, (2) to clarify doubts about content and format of the program and (3) to review and reinforce effort and achievements.

#### 2.3.3. Videoconferencing Treatment

Videoconferencing treatment was delivered weekly by trained therapists, with each session lasting approximately 60 min. The therapeutic components were the same ones used in the GROw group, but explained by a therapist instead of by videos, images, etc. At the end of the session, participants received a summary of the session and the weekly tasks assigned by email. For some sessions (i.e., mindfulness, compassion, and self-compassion) the participants also received meditation audios. This treatment was designed to be carried out through weekly sessions for 8–10 weeks. Participants were instructed to do one session per week, being able to do sessions 4, 5 or 6 (dedicated to writing assignments for reconstructing the meaning of loss) in two weeks due to its complexity and length. No weekly support call was provided in this condition.

### 2.4. Outcome Measures

Participants were assessed with both semi-structured telephone interviews and self-administered questionnaires through a web platform: https://psicologiaytecnologia.labpsitec.es (accessed on 15 July 2025). The evaluation was the same in both groups (GROw and videoconferencing group). The interviews were conducted by experienced clinicians in terms of stress-related disorders. A clinical team of mental health professionals oversaw all the cases. Automatic reminder emails for each assessment were sent to clinicians and participants.

#### 2.4.1. Screening, Diagnostic Measures and Demographics

Some inclusion and exclusion criteria (e.g., internet access) were assessed using an interview that was specifically designed for this study. Demographic data were assessed through phone interviews and included age, sex, civil status, occupation, educational level, and place of residence. The following instruments were selected as diagnostic measures:

Inventory of Complicated Grief (ICG: Limonero et al., 2009; Prigerson et al., 1995). This self-administered questionnaire assesses the frequency of emotional, cognitive, and behavioral symptoms. ICG has 19 Likert-type items with 4 points (from “never” to “always”) and a total ICG score ≥ 25 indicates complicated grief. The ICG has good psychometric properties in both its original version (Cronbach’ alpha coefficient = 0.94 and test–retest reliability = 0.80) (Prigerson et al., 1995) and Spanish adaptation (Cronbach’ alpha coefficient = 0.88 and test–retest reliability = 0.81).

Structured Clinical Interview for Complicated Grief (SCI-CG: Bui et al., 2015). The SCI-CG is a 31-item clinician-administered instrument to assess the relationship with the deceased, cause of death, time since the death (<6 months, between 6 and 12 months, or >12 months) and the presence of complicated grief symptoms. Each of the 31 items is rated on a 3-point Likert-type scale: 1 = not present, 2 = unsure or 3 = present. The SCI-CG has good psychometric properties in its original English version (good internal consistency, interrater and test–retest reliability, and convergent validity in a sample of individuals with complicated grief). The SCI-CG was translated into the Spanish language following a back-translation procedure.

Anxiety Disorders Interview Schedule (ADIS; Di Nardo et al., 1994). The severity rating scale of the ADIS was used to assess the level of distress-interference in functioning. This scale ranges from 0 = absent to 8 = very severely disturbing/disabling.

With the information from the ICG, SCI-CG and the ADIS severity rating scale, a team of clinicians and researchers with experience in stress-related disorders determined whether each participant met the diagnostic criteria for PGD (World Health Organization, 2018).

#### 2.4.2. Primary Outcomes: Measures of Feasibility

Adherence was assessed considering the number of completed sessions/modules, dropout percentages and, in the iCBT format (GROw), time spent on each module and how many times the participants accessed and reviewed the modules.

Preferences and opinion when comparing the two types of intervention formats (GROw vs. videoconferencing treatment) were assessed using a 5-point questionnaire created specifically for this study that evaluated: format preference, subjective efficacy, intervention logic, subjective aversiveness and recommendation to other family members or friends. Preferences were measured before and after treatment. Before treatment, a brief explanation of the content and format of the two treatments (GROw and videoconferencing) was given in both groups.

Expectations and Satisfaction Questionnaire (adapted from Borkovec & Nau, 1972). This questionnaire has 6 items on a scale from 0 = nothing to 10 = very much and assesses opinions about the logic of the intervention, satisfaction with the treatment, willingness to recommend the treatment to family or friends, usefulness for treating other problems, personal usefulness, and aversiveness. This questionnaire was administered before and after the intervention. A brief explanation of the content and format of the treatment was given in both groups before the treatment.

Additionally, several questions about the opinion of the treatment were assessed after the intervention using a semi-structured opinion interview with quantitative and qualitative questions delivered in both conditions: GROw and videoconferencing treatment. This interview included a list of 9 points (e.g., motivation to change, mindfulness, exposure) about the usefulness of each therapeutic content (assessed from 0; not at all useful to 10; maximum usefulness). In addition, for the GROw group, a list of 4 points (text, video, audio and images) about the usefulness of each part of the iCBT format (from 0; not useful at all to 10; maximum usefulness), three semi-structured questions about the opinion of the weekly calls from a therapist (satisfaction and usefulness related to the subjective recovering and the adherence to the treatment on a scale from 0= not at all to 10 = very much), and an open question about unexpected effects (including unexpected effects due to the health crisis produced by COVID-19) were all included.

#### 2.4.3. Psychological and Mental Health Outcomes

Psychological and mental health outcomes were assessed using: the Inventory of Complicated Grief (ICG) (Limonero et al., 2009; Prigerson et al., 1995), the Beck Depression Inventory—Second Edition (BDI-II) (Beck et al., 1996; Estevan et al., 2019), the Typical Beliefs Questionnaire (TBQ) (Skritskaya et al., 2017), the Overall Anxiety Severity and Impairment Scale (OASIS) (Campbell-Sills et al., 2009; González-Robles et al., 2018), the Overall Depression Severity and Impairment Scale (ODSIS) (Bentley et al., 2014; Mira et al., 2019), the Positive and Negative Affect Schedule (PANAS) (Díaz-García et al., 2020; Watson et al., 1988), the Quality of Life Index (QLI) (Mezzich et al., 2000), the Work and Social Adjustment Scale (WSAS) (Echezarraga et al., 2019; Mundt et al., 2002), the Posttraumatic Growth Inventory (PTGI) (Tedeschi & Calhoun, 1996; Weiss & Berger, 2006), the Purpose-In- Life Test (PIL-10) (García-Alandete, 2014), the Five-Facet Mindfulness Questionnaire (FFMQ-15) (Asensio-Martínez et al., 2019) and the Self-Compassion Scale-Short Form (SCS-SF) (Garcia-Campayo et al., 2014; Raes et al., 2011). The time of assessment, source of measurement and assessed group can be consulted in the study protocol (Tur et al., 2021).

### 2.5. Statistical Analysis

As the main aim of this study is related to feasibility, the data has been reported narratively, illustrated using the CONSORT 2010 statement (Eldridge et al., 2016) to guide the reporting of this information. One-way ANOVA for continuous data and chi-square tests for categorical variables were used to verify significant differences between groups in sociodemographic measures. Shapiro–Wilk tests were conducted to verify the normality of the sample distribution.

Means, standard deviations and percentages/frequencies were used to explore feasibility measures: preferences and opinions when comparing the two types of intervention formats (GROw vs. videoconferencing), expectations, satisfaction, and participant’ opinion about the usefulness of the intervention. Non-parametric analyses were used to explore significant differences between groups and pre-post treatment changes in these feasibility measures. The chi-square test was used to explore differences between groups related to preferences and opinions when comparing the two types of intervention formats. Mann–Whitney’s U tests were conducted to explore differences between the groups related to expectations, satisfaction, and participants’ opinion about the usefulness of the intervention. Wilcoxon tests were carried out to explore significant changes between the expectations (pre-treatment) and satisfaction (post-treatment) in both groups. Attrition and dropout rates were also calculated to explore feasibility by reporting percentages and patterns of missing data. In the GROw group, the number of times each patient used the program was used as the measure of adherence. In the videoconferencing group, the number of times each patient attended the sessions was used as the measure of adherence. In addition, means and standard deviations were used to explore adherence to GROw, considering the number of times each patient reviewed each program module and the time spent in each one.

As a secondary objective, the potential effectiveness of iCBT was assessed by applying within-group mixed model analyses between baseline and post-treatment for ITT and within-group t-tests for completers data. In addition, effect sizes (Cohen, 1988) and their respective 95% CI for intragroup changes were reported. Comparisons between iCBT and videoconferencing CBT were not accomplished. Sensitivity analyses were conducted to ensure the robustness of the applied statistical methods and their adequacy (Thabane et al., 2013; Viel et al., 1995). Compared to the statistical plan proposed in the published protocol of this study (Tur et al., 2021), mixed-model analyses were conducted for ITT to increase the data available for exploring the potential efficacy based on author recommendations and due to the large amount of missing data. Follow-ups were not included in the analysis due to the large number of missing values. ITT analyses are reported in the results section, and completers data are attached as Appendix A. The IBM SPSS Statistics (V.25) was used for all statistical analysis.

## 3. Results

### 3.1. Baseline and Participant Characteristics

Participants’ sociodemographic information is presented in Table 1. No significant differences were found at baseline. Overall, the main age of the sample was 39.13 (SD:12.57) and participants were mostly women (93.5%).

### 3.2. Feasibility Results

#### 3.2.1. Participants Recruitment Process and Adherence

**Figure 2:**
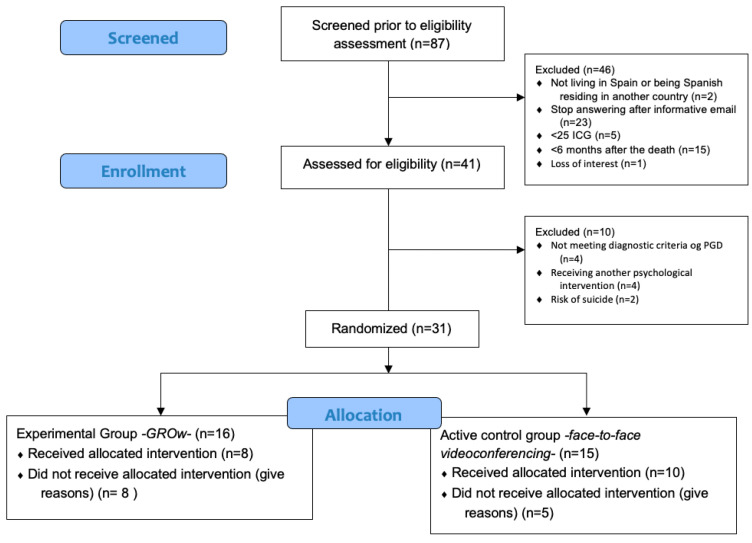
Participants flow diagram.

Recruitment of participants began in December 2020 and ended in April 2022. A flow chart of the participant recruitment is presented in Figure 2. Eighty-seven people were interested in participating in the study and 41 of them completed a telephone evaluation by a professional with experience in stress and trauma-related disorders. Of these participants, 31 met all the inclusion/exclusion criteria of the study and were randomly assigned to the GROw group (N = 16) and the face-to-face videoconferencing group (N = 15). According to the recruitment process, 38.7% of the participants found out about the study from other people who informed them, 35.5% found out about the study through social networks (Twitter and Instagram), 16.1% through local media (newspaper), 6.5% by seeing posters placed at Universitat Jaume I, and 3.2% were patients at the Emotional Disorders Clinic at Universitat Jaume I. Thirteen people (41.9%) dropped out of the study before completing their treatment: 8 participants in the GROw group and 5 participants in the videoconferencing group. The dropout rate was higher in the GROw group (50%) compared to the videoconferencing group (33.3%).

In the GROw group, three people (18.70%) dropped out of treatment before completing module 1, two people (12.5%) after completing module 3, two people (12.5%) after completing module 4, and one person (6.3%) dropped out after completing module 5. In the videoconferencing group, none of the 5 participants who dropped out of the study completed the first treatment session, dropouts occurred after randomization and before starting the intervention.

In the GROw group, the mean number of weeks to finish treatment was 16.86 (118 days; SD = 51.4). In the videoconferencing group, the mean number of weeks to complete the treatment was 10.70 (75.38 days; SD = 8.33).

Table 2 shows the number of times that participants in the GROw group accessed and reviewed the modules, and the mean time spent in each one.

#### 3.2.2. Preferences and Opinions When Comparing the Two Types of Intervention Formats

Table 3 shows participants’ preferences about the type of treatment (GROw and videoconferencing) and their opinions about which one they thought was more effective, logical, aversive, and recommended. Preferences were requested before and after the intervention.

Overall, a greater number of participants chose videoconferencing treatment instead of GROw. In addition, participants perceived videoconferencing treatment as more effective, logical, and recommended for a friend or family member. Before treatment, a greater number of participants chose videoconferencing treatment as more aversive. After treatment, GROw participants perceived this treatment as more aversive.

The chi-squared test revelated no statistically significant differences between groups before treatment. After treatment, statistically significant differences were obtained in three variables: preferences (χ^2^(2) = 9.52, *p* = 0.009), subjective efficacy (χ^2^(2) = 12.99, *p* = 0.002) and recommendation (χ^2^(2) = 17.00, *p* = 0.000).

#### 3.2.3. Expectations and Satisfaction

Both groups reported high satisfaction after treatment (See Table 4).

Results showed high expectations (pre-treatment) and satisfaction (post-treatment) scores for most of the items. The item related to “Aversiveness” showed low to medium mean scores in both GROw and videoconferencing groups. Statistically significant differences were found (Mann–Whitney’s U, *p* < 0.05) between groups in two variables pre-treatment (expectations): intervention logic (higher score for videoconferencing treatment) and treatment recommendation (higher score for GROw treatment). No statistically significant differences were found (Mann–Whitney’s U, *p* > 0.05) between groups post-treatment (satisfaction).

Considering both GROw and videoconferencing groups, Wilcoxon tests showed no statistically significant differences regarding the expectations and satisfaction from pretreatment to post-treatment (*p* > 0.05 in all variables). Specifically for each group, Wilcoxon tests showed a statistically significant mean increase in the GROw group from pretreatment to post-treatment for the intervention logic variable (z = −2.01, *p* = 0.038) and a statistically significant decrease in videoconferencing intervention from pre-treatment to post-treatment in the usefulness for treating other problems variable (z = −2.23, *p* = 0.026).

#### 3.2.4. Qualitative Interview

Results showed that, on average, the participants perceived the therapeutic components of the treatment as useful in both groups, with a mean score greater than 7.5 (0 = not useful; 10 = very useful) in all components (Table 5). In the GROw group, the components that obtained the highest score were experiential exposure and the writing assignments, while in the videoconferencing group, the component that was perceived as most useful was the one with the writing assignments. No significant differences were found between the means of the groups for all variables related to the usefulness of therapeutic components (Mann–Whitney’s U, *p* > 0.05 in all comparisons).

Only the participants of the GROw group answered the questions about the different elements of the web platform. On average, the participants perceived all elements (images, videos, audios, and written information) as useful with a mean score greater than 8.5 (0 = not useful; 10 = very useful) in all of them. Written information was the best valued element with an average of 9 (0 = not useful; 10 = very useful).

On average, participants in the GROw group perceived the weekly follow-up phone calls as useful and expressed high satisfaction with the phone calls (see Table 6). In addition, their qualitative opinion of the calls was recorded. Comments included “the call was a connection between the work I was doing and listening to a voice that understood me and answered my questions, asking me how this week was. Not feeling detached and alone with the computer screen, but a person with whom to have a chat”, “it helps you move forward; it pushes you to keep up, it gives you support and confidence in case you have doubts”, “useful to answer all questions. Being able to talk and get feedback. I tried to do a module every week and it helped me a lot to understand why I was not being able to continue with an exercise or what was happening with me emotionally in relation to the module”, “it makes you not completely disconnect, you have an obligation, and the support of the person, you can ask questions, if you have any problem, you have that person.”

An open question about unexpected effects (including unexpected effects due to the health crisis produced by COVID-19) was included and no one in either of the GROw or videoconferencing group reported adverse effects.

Results of the ITT pre- to post-treatment comparisons by means of linear mixed models for the videoconferencing group showed statistically significant differences with large effect sizes (d > 0.50) (Norcross & Lambert, 2018) for all outcomes except for ODSIS, WSAS, FFMQ-15 and SCS-SF (See Table 6).

For the GROw group, ITT pre- to post-treatment comparisons show statistically significant differences with large effect sizes (d > 0.50) (Norcross & Lambert, 2018) for all outcomes except for WSAS, FFMQ-15 and SCS-SF (See Table 7).

Results of the completers analysis for both GROw and videoconferencing groups are included as Appendix A.

## 4. Discussion

This feasibility trial found that GROw, an iCBT for people with PGD, was acceptable for participants compared to the same intervention delivered through videoconferencing. Also, the preliminary efficacy data showed significant symptom reduction.

### 4.1. Recruitment

As for the recruitment of participants, it lasted 2 years and 4 months and the number of participants who entered the study was lower than expected (31/48) in the published study protocol (Tur et al., 2021). The prevalence of PGD is one out of ten in bereaved adults (Lundorff et al., 2017) worldwide and, in Spain, it accounts for 7.67–10.68% of people who have lost a loved one. The prevalence of PGD is lower compared to other psychological disorders, such as anxiety disorders and mood disorders, which represent 20–28% of the total population, with comorbidity rates ranging from 40 to 80% (Kessler et al., 2005). This may be one reason behind the difficulty of recruiting a larger number of participants. In addition, according to ICD-11, at least 6 months must pass since the death of the loved one to make the diagnosis of PGD. In this regard, 17.3% of the individuals who reached out to participate in the study were directly excluded according to this exclusion criteria. Regarding recruitment strategies, most participants learned about the study through others who informed them and, to a lesser extent, through social media, local media, posters at the Universitat Jaume I, and the Emotional Disorders Clinic at the same university. In this sense, exclusively online recruitment strategies may not fully represent the general population (Glasgow et al., 2007; Whitehead, 2007). Attempts to collaborate with other clinical centers and bereavement associations were unsuccessful. Future studies could benefit from collaboration with clinical centers to expand the sample size and improve its representativeness.

### 4.2. Adherence and Dropouts

According to the adherence data, the mean number of weeks to finish the treatment was 16.86 in the GROw group (118 days; SD = 51.4), while in the videoconferencing group the mean number of weeks to complete the treatment was 10.70 (75.38 days; SD = 8.33). Participants in the videoconferencing group were more closely monitored by a therapist, and this may be a reason why the time spent undergoing the treatment was more aligned with the planned timing. The treatment modules in the GROw group were designed to take approximately one hour, but the results indicated that the participants needed an average ranging from 317 to 568 min (depending on the treatment module) to complete the module. According to the literature, the mean number of treatment sessions for disturbing or disabling grief in other published Internet-based intervention is 10 with a range of 1 to 20 (Johannsen et al., 2019). Treatments with good efficacy results such as the “Complicated Grief Treatment” (CGT) (Shear, 2015a) require 16 weeks of intervention. This is closer to the time used in the GROw treatment. Future research should take this into account to adjust the timing of the treatment. For example, for a blended CBT intervention, 10–12 days (per module) could be considered to review information and do the exercises through the web platform after having a brief session with a therapist.

We defined dropout as those participants who were randomized and assigned to a group but did not access or complete treatment. A little less than half of the participants (41.9%) dropped out of the study (50% in the GROw group and 33.3% in the videoconference group). This is consistent with the results obtained in other studies of Internet-based treatments for grief, which found that rates of dropouts ranged from 10.3 to 58.8%. This is also consistent with a preliminary study that evaluated the effect of GROw in the reduction of symptoms through a multiple-baseline single-case experimental design study in which 34% of participants dropped out of the GROw treatment (Tur et al., 2022).

### 4.3. Preferences and Opinions

According to patients’ preferences and opinions when comparing the two types of intervention formats before treatment, in general, a greater number of participants chose videoconferencing treatment instead of GROw, and videoconferencing treatment was perceived as more effective, logical, and recommended for a friend or family member. Most participants in the GROw group preferred in-person videoconferencing before treatment. However, at the end of treatment, the majority of those who completed the GROw program preferred it to in-person videoconferencing. In the in-person videoconferencing group, most participants initially preferred this modality, but by the end of treatment, almost all preferred it to the GROw program. These results are not in line with previous studies that conclude that treatment preferences remain stable over time (Delevry & Le, 2019). Overall, the data obtained in this study shows that the preferences and opinions when comparing the two types of intervention formats generally improve in favor of GROw once this treatment is completed. It is important to note that in the GROw group the dropout rate was 50%, while the videoconference group had a 33% drop-out rate. Patients’ preference may be associated with the start of treatment and adherence (Brenes et al., 2021; Delevry & Le, 2019; Raue et al., 2009). Although more studies are needed, the results of this study indicate that there is a tendency to choose face-to-face treatments (in this case using videoconference), although once the iCBT (GROw) has been tested, this tendency changes in favor of the iCBT. Therefore, it is necessary to improve the dissemination and information about these types of treatments and how these interventions can help people with PGD.

### 4.4. Expectations, Satisfaction, and Participants’ Evaluation About the Usefulness of the Intervention

With regard to expectations and satisfaction with the treatment, participants showed high expectations and satisfaction scores for most items in both groups. These results are consistent with other studies that have observed high expectations before treatment (Botella et al., 2016; Campos et al., 2018; Mor et al., 2022) and satisfaction after treatment (Botella et al., 2016; Campos et al., 2018; McGoron et al., 2019; Mor et al., 2022; Palacios et al., 2018; Richards et al., 2016; Seitz et al., 2014) with Internet-based treatments. However, significant differences were found between groups in this regard. First, the videoconference group considered their condition more logical only before treatment, given that no differences were found between groups at post-treatment. This result is consistent with other Internet-based treatment studies in which the participants’ opinion about the treatment logic increases after the intervention is completed (Botella et al., 2016). Second, the GROw group would recommend their treatment condition more to their friends and relatives. This finding is also consistent with other studies that have found high scores related to the recommendation of Internet-based programs over more traditional options (Botella et al., 2016; Campos et al., 2018; Mor et al., 2022). Finally, a significant decrease in the videoconferencing group from pretreatment to post-treatment related to the variable “usefulness for treating other problems” was found. This could be because, despite being an intervention delivered by a therapist, there was a guide for each session that was clearly focused on the specific treatment of PGD.

Regarding the usefulness of the therapeutic content, both groups rated it as very useful. The GROw group particularly valued the experiential exposure, writing assignments, and cognitive reappraisal, while the in-person videoconference group rated the writing assignments, cognitive reappraisal, and compassion strategies as most useful. Experiential avoidance, behavioral avoidance, and expressive suppression are closely linked to distressing grief symptoms (Eisma & Stroebe, 2021; Williams et al., 2019). Therapeutic components such as experiential exposure and writing tasks help patients confront situations, memories, and emotions they have been avoiding. Participants’ positive perceptions of the helpfulness of these components are consistent with findings from other studies incorporating exposure strategies, which have shown promising results in symptom reduction in both in-person and online treatments (Johannsen et al., 2019; Wagner et al., 2020). Cognitive reappraisal and compassion strategies, highly rated as helpful by participants, likely provided effective emotion regulation tools. Adaptive emotion regulation strategies, including cognitive reappraisal and mindfulness, are generally associated with a reduction in distressing grief symptoms (Eisma & Stroebe, 2021). Studies integrating cognitive reappraisal into internet-based treatments have also demonstrated positive results in reducing grief-related symptoms (Hoffmann et al., 2018; Kersting et al., 2013; van der Houwen et al., 2010; Wagner et al., 2006). Furthermore, compassion-based therapies, such as Compassion-Focused Therapy (CBT) or The Attachment-Based Compassion Therapy (ACBT, García-Campayo et al., 2023), improve emotional resilience, helping people better tolerate distress and process painful emotions, which can contribute to rebuilding a life after the loss of a loved one (Harris, 2021). Despite the growing interest in the therapeutic components, patients’ subjective experiences regarding these treatments remain underexplored. Further research is essential to better understand this area and develop treatments for people with PGD that not only relieve symptoms but also promote patient satisfaction and adherence.

Participants highly valued the different elements available on the web platform, such as images, videos, audios, and written content, with written information being the most appreciated. This may be attributed to the fact that most of the content was presented in written format, allowing participants to access and review it at their convenience. Few studies have specifically examined the usefulness of each component in online interventions. McGoron et al. (2019) explored an online intervention to promote positive parenting and found that participants valued the clarity, usefulness, and organization of the information. Future research should focus on identifying which features of each element are most effective and satisfying for users. Furthermore, participants in the GROw group also positively valued the weekly follow-up telephone sessions with a therapist. The involvement of a human therapist and the perception of genuine care have been identified as key factors in adherence to online interventions (Andersson, 2009; Christensen et al., 2009; Johansson et al., 2015; Spek et al., 2007). The qualitative findings of our study support this claim, as participants reported that weekly telephone support was beneficial and improved their adherence. Similar conclusions were drawn in other qualitative studies, such as that of Mor et al. (2022), highlighting the importance of therapeutic follow-up in online interventions.

Overall, the results obtained in this study related to the expectations, satisfaction and participants’ evaluation about the usefulness of the intervention are consistent with those of a preliminary study that explored the feasibility of GROw through a multiple-baseline single-case experimental design study with 6 participants (Tur et al., 2022). In that study, participants who completed the treatment (66%) reported high levels of satisfaction with GROw and found both the therapeutic content and the follow-up phone calls to be highly useful.

### 4.5. Potential Efficacy

In line with the secondary aim of the study and based on the collected data, promising results were found regarding the potential efficacy of the treatment in both groups. Both groups showed significant reductions in grief symptoms, anxiety, depression, and maladaptive beliefs about loss, with large effect sizes. Improvements were also observed in positive affect, quality of life, post-traumatic growth, and purpose in life. These findings suggest that both interventions were effective in enhancing emotional well-being and adaptation to loss. This aligns with a preliminary study evaluating the impact of GROw on symptom reduction using a multiple-baseline single-case experimental design (Tur et al., 2022). However, no significant changes were observed in working and social adjustment, mindfulness skills, or self-compassion, which may be attributed to the nature of the intervention and the timeframe in which changes were measured. Although GROw includes mindfulness and self-compassion exercises, its primary focus is on addressing grief-related outcomes, which may explain the significant improvements in depression, anxiety, and emotional affect, but not in social functioning, mindfulness, or self-compassion. These aspects often require more focused interventions, and changes in these areas may take longer to manifest. Our findings suggest that psychological interventions tend to have more immediate effects on emotional distress, while improvements in broader domains occur gradually as participants integrate learned strategies into their daily lives. Future studies with long-term follow-ups are recommended to further explore this.

The GROw group showed slightly higher effect sizes in some of the measures compared to the videoconferencing intervention in the within-group analyses. These results are consistent with the available literature showing that Internet-based treatments for the prevention and treatment of PGD offer promising results in reducing bereavement-related symptoms (Tur et al., 2019; Wagner et al., 2020). It is important to note that the total time of the intervention was longer in the GROw group (Mean = 16.86 weeks) than in the videoconferencing group (Mean = 10.70 weeks). The effect of time was not controlled in this study and could be an important variable for improving grief-related symptoms given that grief symptoms can decrease naturally over time (Jordan & Litz, 2014). The exploratory results about the preliminary efficacy of this study should be taken with extreme caution and more studies are needed. These conclusions cannot be generalized to the general population.

### 4.6. Limitations

This study presents several limitations that must be highlighted. First, the SCI-CG (Bui et al., 2015) used for the diagnosis of PGD was not validated in Spanish population, although it did show adequate psychometric properties in its original version and was back-translated for this study. To ensure an adequate diagnosis, the results of this interview had to be complemented by exceeding the ICG cut-off point in its Spanish version (Limonero et al., 2009). Second, the unequal gender distribution in the sample (with only two men included) could limit the generalizability of the results. Also, the fact that nearly 40% of patients were recruited through recommendations from other study participants may introduce a bias and could overestimate acceptability. Another limitation is related to the sample size. A smaller sample size and higher dropouts were obtained than proposed in the protocol of this study (Tur et al., 2021). For this reason, the results have been obtained from a smaller sample than expected and no follow-ups could be included. Moreover, the influence of the passage of time without intervention was not measured. For all of this, the results about the potential effect of the treatment obtained in this study must be taken with caution. Finally, additional changes made in relation to the published protocol of this study (Tur et al., 2021) and not explained in the method section, should be mentioned: (1) considering that there was already extensive and sufficient information related to the feasibility of this study, the data obtained in the Usability and Acceptance Questionnaire (Campos et al., 2018; Castilla et al., 2016) and the Working Alliance Inventory (WAI) (Horvath & Greenberg, 1989) were not included in this manuscript; (2) as the sample size was estimated to conduct within-group comparisons, between-group analyses were not considered and; (3) due to the pandemic situation caused by COVID-19 and related to the mobility restrictions, the face-to-face sessions were not conducted in person and were finally carried out using videoconference.

## 5. Conclusions

The results of this study show that GROw is a feasible well-accepted iCBT for the treatment of PGD with promising results related to its potential efficacy in terms of reducing grief-related symptoms. GROw shows strong potential as an iCBT for PGD and can be a cost-effective alternative to face-to-face interventions. iCBTs are cost-effective, cheaper in relation to face-to-face therapy, can reach individuals who need therapy and are a well-established therapy format showing clinical efficacy compared to control groups and active conditions in the short and long term (Andersson et al., 2014, 2017; Carlbring et al., 2018; Cuijpers et al., 2008; Donker et al., 2015; Hedman et al., 2011; Karyotaki et al., 2018; Komariah et al., 2022; Musiat & Tarrier, 2014; Wang et al., 2020; Xiong et al., 2022). Furthermore, this form of therapy can overcome geographical barriers and isolation problems (F. Griffiths et al., 2006; K. M. Griffiths & Christensen, 2007) such as those experienced due to COVID-19 which made disturbed grief a major public health concern worldwide (Eisma et al., 2020).

This study supports scaling up the treatment using larger samples and more complex designs such as randomized controlled trials comparing GROw with active controls and including non-active control groups to evaluate the influence of the passage of time without intervention. Our findings also further endorse including more human support in the treatment, for example, blending online self-applied modules with brief face-to-face or videoconferencing CBT sessions. Future studies exploring alternative intervention formats that increase treatment adherence are needed to reach more individuals with PGD.

## 6. Patents

A software titled GROw (Invention Disclosure No. 3024) has resulted from the work reported in this manuscript. It was registered as intellectual property on 13 March 2024, in Spain. The inventors are Quero, S. (50%), Campos, D. (30%), García Palacios, A. (10%), Castilla, D. (5%), and Zaragozá, I. (5%). Ownership is held by Universitat Jaume I (60%), Universidad de Zaragoza (30%), and Universidad de Valencia (10%).

## Figures and Tables

**Table 1:** Participants’ sociodemographic information.

	Total (N = 31)	GROw (N = 16)	Videoconferencing (N = 15)	Between-Group Comparison
**Gender**				χ^2^(1) = 2.28, *p* = 0.131
Female	29 (93.5%)	16 (100%)	13 (86.7%)	
Male	2 (6.5%)	0 (0%)	2 (13.3%)	
**Age mean (SD)**	39.1 (12.6)	37.6 (12.2)	40.7 (13.2)	F (1,29) = 0.465, *p*= 0.501
**Educational level**				χ^2^(2) = 2.36, *p* = 0.307
Primary education	2 (6.5%)	2 (12.5%)	0 (0%)	
Secondary education	12 (38.7%)	5(31.3%)	7 (46.7%)	
Higher education	17 (54.8%)	9 (56.3%)	8 (53.3%)	
**Relationship with the deceased**				χ^2^(5) = 3.23, *p* = 0.665
Grandfather/grandmother	5 (16.1%)	3 (18.8%)	2 (13.3%)	
Father/mother	17 (54.8%)	9 (56.3%)	8 (53.3%)	
Brother/sister	4 (12.9%)	2 (12.5%)	2 (13.3%)	
Partner	2 (6.5%)	1 (6.3%)	1 (6.7%)	
Son/daughter	2 (6.5%)	0 (0%)	2 (13.3%)	
Uncle/aunt	1 (3.2%)	1 (6.3%)	0 (0%)	
**Time since loss**				χ^2^(3) = 1.37, *p* = 0.713
>6 months	9 (29%)	6 (37.5%)	3 (20%)	
12–24 months	10 (32.3%)	5 (31.3%)	5 (33.3%)	
24–48 months	2 (6.5%)	1 (6.3%)	1 (6.7%)	
>24 months	10 (32.3%)	4 (25%)	6 (40%)	
**Type of death**				χ^2^(3) = 2.00, *p* = 0.571
Illness	27 (87.1%)	14 (87.5%)	13 (86.7%)	
Natural death	1 (3.2%)	1 (6.3%)	0 (0%)	
Suicide	2 (6.5%)	1 (6.3%)	1 (6.7%)	
Accident	1 (3.2%)	0 (0%)	1 (6.7%)	

**Table 2:** GROw group participant module access and reviews.

	N	Module Access Mean (SD)	Reviews Mean (SD)	Time (Min) Mean (SD)
Module 1	13	2.2 (1.7)	0.92 (1.0)	340.8 (104)
Module 2	13	2.6 (3.9)	0.54 (1.0)	358.2 (126.4)
Module 3	13	3.1 (5.5)	0.46 (1.0)	364.3 (207.3)
Module 4	11	8.7 (9.9)	0.45 (0.9)	351.3 (141.8)
Module 5	9	2.7 (4.0)	0.11 (0.3)	317.2 (157.4)
Module 6	8	7.4 (10.1)	0.13 (0.4)	321.6 (186.5)
Module 7	8	3.9 (6.0)	0.25 (0.7)	568.8 (276.2)
Module 8	8	1.6 (2.1)	0.13 (0.4)	374.3 (197.3)

**Table 3:** Participants’ preferences about the treatment before and after the intervention.

	Before Treatment (n = 30)		After Treatment (n = 16)	
	GROw (n = 16)	Videoconferencing (n = 14)	GROw (n = 5)	Videoconferencing (n = 11)
Preference				
GROw	7 (41.2%)	5 (35.7%)	4 (80%)	1 (9.1%)
Videoconferencing	9 (52.9%)	9 (64.3%)	1 (20%)	10 (90.9%)
Subjective efficacy				
GROw	3 (17.6%)	4 (28.6%)	4 (80%)	0 (0%)
Videoconferencing	13 (76.5%)	10 (71.4%)	1 (20%)	11 (100%)
Logic				
GROw	2 (11.8%)	2 (14.3%)	1 (20%)	0 (0%)
Videoconferencing	14 (82.4%)	12 (85.7%)	4 (80%)	11 (100%)
Subjective aversiveness				
GROw	4 (23.5%)	6 (42.9%)	5 (100%)	4 (36.4%)
Videoconferencing	12 (70.6%)	8 (57.1%)	0 (0%)	7 (63.3%)
Recommendation				
GROw	4 (23.5%)	4 (28.6%)	5 (100%)	0 (0%)
Videoconferencing	12 (70.6%)	10 (71.4%)	0 (0%)	11 (100%)

**Table 4:** Participants’ expectations and satisfaction with the intervention.

	GROw		Videoconferencing	
	Pretreatment Expectations M (SD) (n = 14)	Post-Treatment Satisfaction M (SD) (n = 5)	Pretreatment Expectations M (SD) (n = 14)	Post-Treatment Satisfaction M (SD) (n = 11)
Intervention logic	7.7 (1.7)	9.4 (0.9)	9.1 (1.0)	9.2 (1.1)
Treatment satisfaction	8.6 (1.6)	9 (1)	9.1 (1.5)	8.8 (1.2)
Treatment recommendation	9.4 (0.9)	10 (0)	9.3 (1.2)	9.5 (0.8)
Usefulness for treating other problems	8.1 (1.8)	9 (1)	9.3 (0.9)	7.9 (1.8)
Personal usefulness	8.1(1.8)	9 (0.7)	9.1 (1.2)	8.6 (1.1)
Aversiveness	5.3 (3.5)	3 (4.1)	4.8 (3.4)	5.1 (3.7)

**Table 5:** Participants’ ratings qualitative interview about usefulness of therapeutic components, usefulness of GROw program characteristics and usefulness/satisfaction with the weekly follow-up phone calls.

	GROw (n = 4) M (SD)	Videoconferencing (n = 10) M (SD)
Usefulness of motivation to change	8.25 (1.26)	7.9 (1.91)
Usefulness of psychoeducation	8.25 (1.71)	8.5 (1.35)
Usefulness of behavioral activation	8.50 (1.29)	8.3 (1.16)
Usefulness of experiential exposure	9.50 (0.58)	8.4 (1.84)
Usefulness of mindfulness	8.25 (0.50)	8.1 (1.73)
Usefulness of compassion strategies	7.50 (1.73)	8.6 (1.08)
Usefulness of writing assignments	9.50 (0.58)	8.9 (1.2)
Usefulness of cognitive reappraisal	9.25 (0.96)	8.8 (1.4)
Usefulness of relapse prevention	8.25 (1.5)	8.5 (1.08)
Usefulness of images	8.50 (1.73)	-
Usefulness of videos	8.75 (0.96)	-
Usefulness of audios	8.75 (0.96)	-
Usefulness of written information	9 (0.82)	-
Satisfaction with the weekly follow-up phone calls	9.50 (1)	-
Usefulness of weekly follow-up phone calls	9 (1.4)	-
Usefulness for adherence of weekly follow-up phone calls	9 (2)	-

**Table 6:** Results of the intent-to-treat pre-to-post comparisons by means of Linear Mixed Models for videoconferencing group.

Outcome	Pre		Post		F	df1	df2	*p*	d
	Mean	SE	Mean	SE					
IGC	41.43	3.98	31.98	4.18	12.23	1	7.67	0.009	1.26
BDI-II	28.86	2.78	15.45	3.07	20.69	1	9.43	0.001	1.48
TBQ	64.03	3.72	47.98	3.97	28.51	1	8.38	<0.001	1.84
OASIS	10.39	1.16	6.66	1.24	16.23	1	8.55	0.003	1.38
ODSIS	9.65	1.23	6.99	1.36	4.22	1	8.76	0.071	0.69
PANAS									
*Positive Affect*	18.10	1.58	22.63	1.67	15.84	1	7.85	0.004	1.42
*Negative Affect*	27.50	2.25	21.05	2.52	6.18	1	9.90	0.032	0.79
WSAS	18.76	2.72	18.38	2.88	0.03	1	8.39	0.858	0.06
QLI	4.39	0.48	5.37	0.49	14.62	1	7.53	0.006	1.39
PTGI	35.49	5.68	48.21	6.16	6.07	1	7.69	0.040	0.89
PIL-10	36.98	2.64	42.48	2.81	7.06	1	7.67	0.030	0.96
FFMQ-15									
*Observe*	2.72	0.23	2.65	0.25	0.22	1	7.72	0.655	−0.17
*Describe*	3.19	0.33	3.14	0.39	0.02	1	8.01	0.895	−0.05
*Awareness*	2.89	0.24	3.01	0.27	0.26	1	8.95	0.624	0.17
*No judgment*	2.69	0.28	3.39	0.33	4.13	1	9.30	0.072	0.67
*No reactivity*	2.72	0.18	2.77	0.20	0.17	1	7.95	0.690	0.15
SCS-SF									
*Self-compassion*	2.37	0.25	2.76	0.27	2.78	1	8.02	0.134	0.59
*Common humanity*	2.77	0.22	2.97	0.25	0.54	1	8.48	0.483	0.25
*Mindfulness*	2.58	0.21	2.39	0.23	0.76	1	8.70	0.407	−0.30

Note. Within-group fixed-effects factor: Time (pretest vs. posttest). SE = Standard error of the mean. F = F-statistic for comparing the pretest–posttest change. df1 and df2 = Degrees of freedom of the numerator and denominator, respectively, of the F-statistic. d = standardized pretest–posttest mean change, calculated by means of: d = √(F/df2). Positive d values indicated a better result in the posttest than in the pretest; and vice versa. Pre = Pretreatment; Post = Post-treatment; M = Mean; SD = Standard deviation; N = Number of participants; BDI-II = Beck Depression Inventory—Second Edition; ICG = Inventory of Complicated Grief; TBQ = Typical Beliefs Questionnaire; PTGI = Post-traumatic Growth Inventory; OASIS = Overall Anxiety Severity and Impairment Scale; ODSIS = Overall Depression Severity and Impairment Scale; PANAS = Positive and Negative Affect Schedule; QLI = Quality of Life Index; PIL-10 = Purpose-In-Life Test; WSAS = Work and Social Adjustment Scale; SCS-SF = Self-Compassion Scale Short; FFMQ-15 = Five-Facet Mindfulness Questionnaire. N = 12.

**Table 7:** Results of the intent-to-treat pre-to-post comparisons by means of Linear Mixed Models for GROw.

Outcome	Pre		Post		F	df1	df2	*p*	d
	Mean	SE	Mean	SE					
IGC	38.00	3.26	19.59	3.99	32.41	1	7.20	<0.001	2.12
BDI-II	23.33	2.33	8.84	2.82	41.28	1	7.51	<0.001	2.34
TBQ	56.20	4.73	37.07	5.55	22.10	1	7.20	0.002	1.75
OASIS	9.47	1.24	4.00	1.43	30.78	1	6.30	0.001	2.21
ODSIS	8.67	1.43	3.90	1.73	12.24	1	7.21	0.010	1.30
PANAS									
*Positive Affect*	19.60	2.07	30.64	2.53	28.90	1	7.71	<0.001	1.94
*Negative Affect*	29.47	1.85	17.42	2.44	27.28	1	8.15	<0.001	1.83
WSAS	16.47	2.56	14.81	3.06	0.49	1	7.43	0.507	0.26
QLI	4.77	0.44	6.78	0.52	30.31	1	6.95	<0.001	2.09
PTGI	30.80	5.96	49.37	7.02	12.81	1	7.34	0.008	1.32
PIL-10	42.87	2.58	54.87	3.42	13.60	1	8.44	0.006	1.27
FFMQ-15									
*Observe*	2.47	0.29	3.06	0.37	3.14	1	8.08	0.114	0.62
*Describe*	3.42	0.26	3.59	0.35	0.23	1	8.05	0.645	0.17
*Awareness*	3.18	0.27	3.58	0.33	1.96	1	6.18	0.210	0.56
*No judgment*	3.51	0.27	3.42	0.28	0.57	1	6.18	0.477	−0.30
*No reactivity*	2.42	0.17	2.54	0.24	0.18	1	10.35	0.682	0.13
SCS-SF									
*Self-compassion*	2.62	0.23	2.92	0.26	3.40	1	6.60	0.110	0.72
*Common humanity*	2.82	0.17	3.28	0.25	3.11	1	11.37	0.105	0.52
*Mindfulness*	2.57	0.17	3.00	0.24	3.05	1	9.75	0.112	0.56

Note. Within-group fixed-effects factor: Time (pretest vs. posttest). SE = Standard error of the mean. F = F-statistic for comparing the pretest–posttest change. df1 and df2 = Degrees of freedom of the numerator and denominator, respectively, of the F-statistic. d = standardized pretest–posttest mean change, calculated by means of: d = √(F/df2). Positive d values indicated a better result in the posttest than in the pretest; and vice versa. Pre = Pretreatment; Post = Post-treatment; M = Mean; SD = Standard deviation; N = Number of participants; BDI-II = Beck Depression Inventory—Second Edition; ICG = Inventory of Complicated Grief; TBQ = Typical Beliefs Questionnaire; PTGI = Post-traumatic Growth Inventory; OASIS = Overall Anxiety Severity and Impairment Scale; ODSIS = Overall Depression Severity and Impairment Scale; PANAS = Positive and Negative Affect Schedule; QLI = Quality of Life Index; PIL-10 = Purpose-In-Life Test; WSAS = Work and Social Adjustment Scale; SCS-SF = Self-Compassion Scale Short; FFMQ-15 = Five-Facet Mindfulness Questionnaire. N = 15.

## Data Availability

The raw data supporting the conclusions of this article will be made available by the authors on request.

## Appendix A

**Table A1:** Results of the completers analysis comparing pretest-post-test mean change for videoconference group.

Outcome	Pre		Post		t	*p*	d
	Mean	SE	Mean	SE			
IGC	45.88	4.53	36.25	5.30	3.48	0.010	1.23
BDI-II	31.75	4.20	17.13	3.39	4.55	0.003	1.61
TBQ	68.00	4.40	51.38	5.87	5.27	0.001	1.86
OASIS	10.88	1.75	7.13	1.68	3.84	0.006	1.36
ODSIS	11.25	1.70	8.50	1.18	1.98	0.088	0.70
PANAS							
*Positive Affect*	17.13	2.12	22.00	2.27	4.16	0.004	1.47
*Negative Affect*	28.00	3.68	21.50	2.25	2.26	0.058	0.80
WSAS	20.63	3.63	20.00	4.64	0.29	0.779	0.10
QLI	3.91	0.64	4.92	0.67	3.84	0.006	1.36
PTGI	28.25	4.60	42.63	7.72	2.72	0.030	0.59
PIL-10	33.13	3.46	39.25	2.64	2.89	0.023	1.02
FFMQ-15							
*Observe*	2.83	0.34	2.75	0.34	−0.55	0.598	−0.19
*Describe*	3.62	0.38	3.33	0.39	−0.70	0.505	−0.25
*Awareness*	3.00	0.33	3.08	0.33	0.31	0.763	0.11
*No judgment*	2.75	0.41	3.42	0.31	1.79	0.117	0.63
*No reactivity*	2.79	0.25	2.83	0.29	0.31	0.763	0.11
SCS-SF							
*Self-compassion*	2.25	0.34	2.66	0.27	1.69	0.135	0.60
*Common humanity*	2.81	0.26	3.03	0.26	0.70	0.456	0.28
*Mindfulness*	2.69	0.30	2.44	0.26	−1.10	0.306	−0.39

Note. Within-group fixed-effects factor: Time (pretest vs. posttest). SE = Standard error of the mean. t = t-statistic for comparing the pretest-posttest change. d = standardized pretest-posttest mean change (Cohen’s d), calculated by means of: $d=\frac{\bar{Y_{Pre}}-\bar{Y_{Post}}}{S_{D}}$ with SD being the change scores standard deviation. Positive d values indicated a better result in the posttest than in the pretest; and vice versa. N = 8.Pre = Pretreatment; Post = Post-treatment; M = Mean; SD = Standard deviation; N = Number of participants; BDI-II = Beck Depression Inventory—Second Edition; ICG = Inventory of Complicated Grief; TBQ = Typical Beliefs Questionnaire; PTGI = Post-traumatic Growth Inventory; OASIS = Overall Anxiety Severity and Impairment Scale; ODSIS = Overall Depression Severity and Impairment Scale; PANAS = Positive and Negative Affect Schedule; QLI = Quality of Life Index; PIL-10 = Purpose-In-Life Test; WSAS = Work and Social Adjustment Scale; SCS-SF = Self-Compassion Scale Short; FFMQ-15 = Five-Facet Mindfulness Questionnaire.

**Table A2:** Results of the completers analysis comparing pretest-post-test mean change for GROw group.

Outcome	Pre		Post		t	*p*	d
	Mean	SE	Mean	SE			
IGC	40.43	3.96	21.43	6.03	5.62	0.001	2.12
BDI-II	24.29	4.29	9.57	3.58	6.16	<0.001	2.33
TBQ	54.71	7.78	35.86	8.65	4.42	0.004	1.67
OASIS	10.14	1.58	4.57	1.64	5.59	0.001	2.11
ODSIS	8.43	2.77	3.71	1.71	3.31	0.016	1.25
PANAS							
*Positive Affect*	17.43	2.86	29.00	4.23	5.28	0.002	2.00
*Negative Affect*	29.71	2.49	17.57	3.19	4.89	0.003	1.85
WSAS	17.43	3.53	15.57	5.17	0.74	0.486	0.28
QLI	4.60	0.85	6.64	0.62	5.38	0.002	2.03
PTGI	35.43	8.84	53.14	12.24	3.24	0.018	1.22
PIL-10	43.57	4.36	55.29	3.81	3.31	0.016	1.25
FFMQ-15							
*Observe*	2.81	0.50	3.29	0.43	1.31	0.237	0.50
*Describe*	3.57	0.37	3.67	0.39	0.26	0.805	0.10
*Awareness*	3.57	0.35	3.86	0.28	1.00	0.356	0.38
*No judgment*	3.62	0.41	3.52	0.39	−0.79	0.457	−0.30
*No reactivity*	2.76	0.21	2.57	0.25	−0.60	0.569	−0.23
SCS-SF							
*Self-compassion*	2.75	0.33	3.03	0.41	1.71	0.139	0.64
*Common humanity*	2.82	0.21	3.28	0.36	1.45	0.197	0.55
*Mindfulness*	2.64	0.25	3.04	0.31	1.39	0.214	0.52

Note. Within-group fixed-effects factor: Time (pretest vs. posttest). SE = Standard error of the mean. t = t-statistic for comparing the pretest-posttest change. d = standardized pretest–posttest mean change (Cohen’s d), calculated by means of: $d=\frac{\bar{Y_{Pre}}-\bar{Y_{Post}}}{S_{D}}$ with SD being the change scores standard deviation. Positive d values indicated a better result in the posttest than in the pretest; and vice versa. N = 8.Pre = Pretreatment; Post = Post-treatment; M = Mean; SD = Standard deviation; N = Number of participants; BDI-II = Beck Depression Inventory—Second Edition; ICG = Inventory of Complicated Grief; TBQ = Typical Beliefs Questionnaire; PTGI = Post-traumatic Growth Inventory; OASIS = Overall Anxiety Severity and Impairment Scale; ODSIS = Overall Depression Severity and Impairment Scale; PANAS = Positive and Negative Affect Schedule; QLI = Quality of Life Index; PIL-10 = Purpose-In-Life Test; WSAS = Work and Social Adjustment Scale; SCS-SF = Self-Compassion Scale Short; FFMQ-15 = Five-Facet Mindfulness Questionnaire.
